# Bacterial Communities of Two Ubiquitous Great Barrier Reef Corals Reveals Both Site- and Species-Specificity of Common Bacterial Associates

**DOI:** 10.1371/journal.pone.0010401

**Published:** 2010-04-29

**Authors:** E. Charlotte E. Kvennefors, Eugenia Sampayo, Tyrone Ridgway, Andrew C. Barnes, Ove Hoegh-Guldberg

**Affiliations:** 1 Centre for Marine Studies, The University of Queensland, Brisbane, Queensland, Australia; 2 Australian Research Council Centre of Excellence for Coral Reef Studies, The University of Queensland, Brisbane, Queensland, Australia; 3 School of Biological Sciences, The University of Queensland, Brisbane, Queensland, Australia; 4 Global Change Institute, The University of Queensland, Brisbane, Queensland, Australia; University of Wisconsin-Milwaukee, United States of America

## Abstract

**Background:**

Coral-associated bacteria are increasingly considered to be important in coral health, and altered bacterial community structures have been linked to both coral disease and bleaching. Despite this, assessments of bacterial communities on corals rarely apply sufficient replication to adequately describe the natural variability. Replicated data such as these are crucial in determining potential roles of bacteria on coral.

**Methodology/Principal Findings:**

Denaturing Gradient Gel Electrophoresis (DGGE) of the V3 region of the 16S ribosomal DNA was used in a highly replicated approach to analyse bacterial communities on both healthy and diseased corals. Although site-specific variations in the bacterial communities of healthy corals were present, host species-specific bacterial associates within a distinct cluster of gamma-proteobacteria could be identified, which are potentially linked to coral health. Corals affected by “White Syndrome” (WS) underwent pronounced changes in their bacterial communities in comparison to healthy colonies. However, the community structure and bacterial ribotypes identified in diseased corals did not support the previously suggested theory of a bacterial pathogen as the causative agent of the syndrome.

**Conclusions/Significance:**

This is the first study to employ large numbers of replicated samples to assess the bacterial communities of healthy and diseased corals, and the first culture-independent assessment of bacterial communities on WS affected Acroporid corals on the GBR. Results indicate that a minimum of 6 replicate samples are required in order to draw inferences on species, spatial or health-related changes in community composition, as a set of clearly distinct bacterial community profiles exist in healthy corals. Coral bacterial communities may be both site and species specific. Furthermore, a cluster of gamma-proteobacterial ribotypes may represent a group of specific common coral and marine invertebrate associates. Finally, the results did not support the contention that a single bacterial pathogen may be the causative agent of WS Acroporids on the GBR.

## Introduction

The successful establishment and survival of coral reefs in tropical nutrient-limited waters is made possible by the symbiosis between two phylogenetically distinct eukaryotes, cnidarians and dinoflagellates of the genus *Symbiodinium*. Reef building corals harbour a highly diverse range but often species-specific community of these intracellular symbionts [1]. Besides this vital symbiosis, corals also sustain highly diverse communities of a broad range of other organisms [2], [3]. Prokaryotes are the most diverse and numerous organisms across all ecosystems [4] and marine archaea as well as eubacteria are associated with reef corals [2], [5], [6]. The finding that coral reef associated prokaryotes are more than just contaminants from the water column [2], [7]–[9] has prompted the establishment of the coral holobiont hypothesis wherein, alongside the important role of *Symbiodinium* in providing the major food source for corals, bacteria, archaea, endolythic algae and fungi all form a functionally relevant mutualistic relationship with reef corals [2], [10].

Unlike *Symbiodinium* which are strictly intracellular, bacteria are found in a range of coral associated micro-niches, including the mucus layer covering coral host tissues [11]–[13], on the coral tissue surface [9], and intracellularly [14]–[16]. Within these bacterial communities, there are several specific groups of bacteria that, to date, are associated with corals [2], [3], [17], and evidence suggests that this specificity extends further to distinct coral hosts [7], [17]. Similar species-specific microbial associations have also been found in sponges and hydra [18]–[20]. Whilst the exact function of many coral-associated bacteria remains unknown, there are indications that certain bacteria provide a food source for coral, either directly or indirectly [16], [21]–[23]; or function in protection against invasive bacteria and other fouling agents [11] by successfully occupying specific niches and through the production of anti-microbial compounds [24]–[28]. Despite the potential importance of coral bacterial associates, very few studies have characterised the differences in the coral bacterial community composition between coral species, locations and biogeographic regions in a replicated and statistically robust manner.

Increased reporting of severity and incidence of both coral bleaching and coral diseases on a global scale [29]–[32], has fueled a plethora of coral bacterial studies, which have predominantly focused on targeting the potential causative pathogens involved in both bleaching and disease syndromes. Potential pathogens implicated in relation to diseased corals include cyanobacterial consortia [29], *Serratia marcescens* [33], *Aurantimonas coralicida* [34], [35], *Vibrio shiloi* [36], [37], *Vibrio coralliilyticus* [38], *Thalassomonas loyana* [39] and an assortment of various *Vibrio* ribotypes [40], [41]. However, despite extensive research into coral disease over the past decade, there is still no consensus on many of the pathogens involved as putative pathogens have not been detected in numerous subsequent studies utilizing culture-independent methods [9], [17], [42]–[46], and the causative agents for most coral disease syndromes remain unknown [47].

Culture-based methods have proven successful to document bacterial community changes on bleached and diseased corals [25], [48]–[50], but the difficulties associated with culturing marine bacteria coupled with a bias towards groups of bacteria that readily grow on general culture media limits the usefulness of such methods in describing bacterial diversity in environmental samples [7], [51], [52]. Culture-independent methods such as fluorescence *in situ* hybridisation (FISH) using specific oligonucleotide probes targeting the 16S ribosomal RNA gene [15], [53], scanning electron microscopy [12], [18], and sequence analyses of the ribosomal 16S region [2], [17], [42], [54] offer an alternative approach. Although factors such as DNA quality [7], primer selection [55], [56], and PCR related biases [57], [58] can potentially confound the outcomes of sequence analyses in terms of species diversity and abundance, the analyses of the ribosomal DNA coupled with restriction fragment length polymorphism (RFLP), denaturing gradient gel electrophoresis (DGGE), or clone library screening still remains the most widely used approach in bacterial community analyses [4], [59].

Culture-independent methods each have advantages and disadvantages (for reviews see [4], [60]) and their underlying differences may have contributed to the reported discrepancies in coral bacterial ecological studies in terms of detecting consistent host-specific bacterial associates (see [2], [7], [17], [61]) or causative disease agents [45], [46], [62], [63]. Clone libraries provide a comprehensive assessment of the microbial community of any given sample, but are usually restricted to a limited number of samples (<5) due to both financial cost and laboriousness of the approach. On the other hand, rapid screening techniques such as RFLP and DGGE allow for larger sample sizes, but their ability to recover all microbial associates present within a sample is reduced compared to clone libraries. Nonetheless, DGGE has been successfully employed in assessments of microbial communities in environmental samples over space and time (e.g. bacterial succession, [64]; waste water treatment, [65]; microbial mats, [66], [67]) and has gained popularity in sponge and coral bacterial studies over the last few years (e.g. [7], [17], [19], [54], [61], [68]).

**Figure 1:**
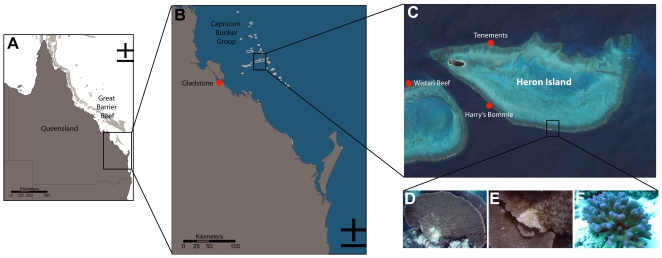
Study sites. Study area and sites in relation to region (A), southern Great Barrier Reef (B), Heron Island and Wistari Reef (C). D–F are representative images of corals sampled: apparently healthy *Acropora hyacinthus* (D), *Acropora hyacinthus* affected by White Syndrome (E) and apparently healthy *Stylophora pistillata*. Images are from NASA/Goddard Space Flight Centre Scientific Visualization Studio, E. Sampayo and G. Roff.

Despite recent research on coral bacterial communities driven by increased reporting of coral diseases and syndromes, reef-wide patterns of naturally (healthy) occurring communities remain largely un-characterized (but see [2], [3], [7], [69]). In general, sample sizes have been limited for coral associated bacterial community analyses, which is problematic if variation occurs between individuals within a population [3]. A detailed understanding of the healthy coral bacterial community is imperative if we are to understand their roles in the coral holobiont and coral disease ecology. To address this, the present study employs a highly replicated approach utilising DGGE to assess the bacterial communities of two common coral species, *Acropora hyacinthus* and *Stylophora pistillata*, sampled across three locations on a single reef system around Heron Island (Great Barrier Reef – GBR, Figure 1). The data provide insight into coral species-specific bacterial associates, documents the natural variation of bacterial communities on corals over small spatial scales, and is indicative of bacterial species interactions on coral reefs. In addition, the inclusion of ‘White Syndrome’ [41], [63], [70]–[72] affected *A. hyacinthus* colonies in the study revealed disease related changes in the bacterial community.

## Results

A total of 134 different bands were identified on the DGGE gels, where coral samples (n = 69) and water (n = 12) produced a total of 124 and 10 bands, respectively. Bands occurring in several replicate samples as well as bands occurring only once but with high band intensity, or bands unique to diseased samples were sequenced (62 bands in total) and deposited in the GenBank database with the accession numbers GQ924692–GQ924753. No chimeric sequences were detected.

### Water associated bacterial communities

Principal component analyses (PCA) showed that the bacterial communities associated with *Acropora hyacinthus*, *Stylophora pistillata*, and the surrounding reef water were significantly different from each other (R = 0.975, p = 0.001; with each subsequent pairwise comparison having a p = 0.001). The bacterial community composition of all water samples was relatively consistent (83% average similarity, SIMPER), but site-specific differences were evident (R = 0.700, p = 0.01). Bacterial community DGGE profiles from water collected at Tenements and Wistari Reef were identical, whilst water samples from Harry's Bommie were identical within replicate samples but differed significantly (p = 0.03) and were more diverse (H′ = 2.303) in comparison to Tenements and Wistari Reef (H′ = 2.079). Sixty percent of the water associated bacterial groups comprised of unknown α-proteobacteria, with the remaining 40% belonging to the *Cythophaga-*, *Flavobacterium-*, *Bacteroides* (CFB) group and the *Rhodobacteraceae* (Table S1). Certain water-associated bacteria were found in coral samples of *A. hyacinthus* (represented by bands W1, W2, and W7; Table S1) and *S. pistillata* (band W8; Table S1), although these were not abundant within or across samples (occurring in <3 individuals) and are likely to represent contaminants from the water column.

### Coral associated bacterial communities

**Figure 2:**
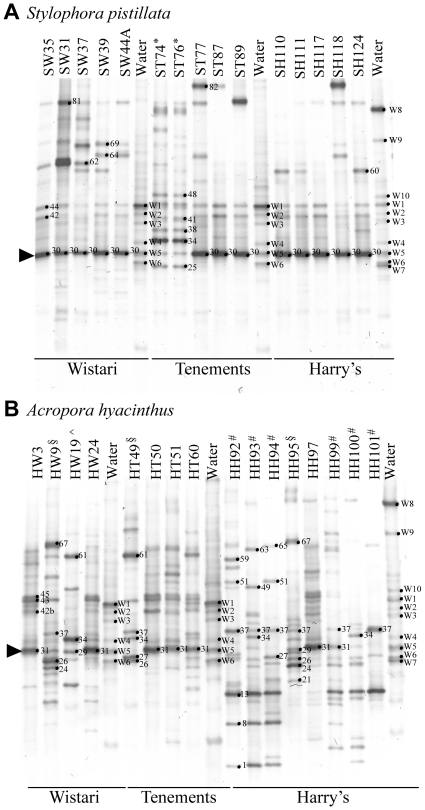
Representative denaturing gradient gel electrophoresis (DGGE) gels of bacterial community profiles in healthy corals and water. DGGE gels of the 16S rDNA hypervariable region V3 show the various bacterial communities present colonies of (A) *Stylophora pistillata* and (B) *Acropora hyacinthus*. Water samples and representative profiles of all profile types from all sites (Wistari Reef, Tenements, Harry's Bommie) are included on both gels. Sample numbers are shown above each lane and band numbers indicate the position of bands excised for sequence analyses. *, #, ^∧^, § identify samples specifically included because they show significant deviations from the most commonly observed bacterial communities as seen in the majority of samples.

**Figure 3:**
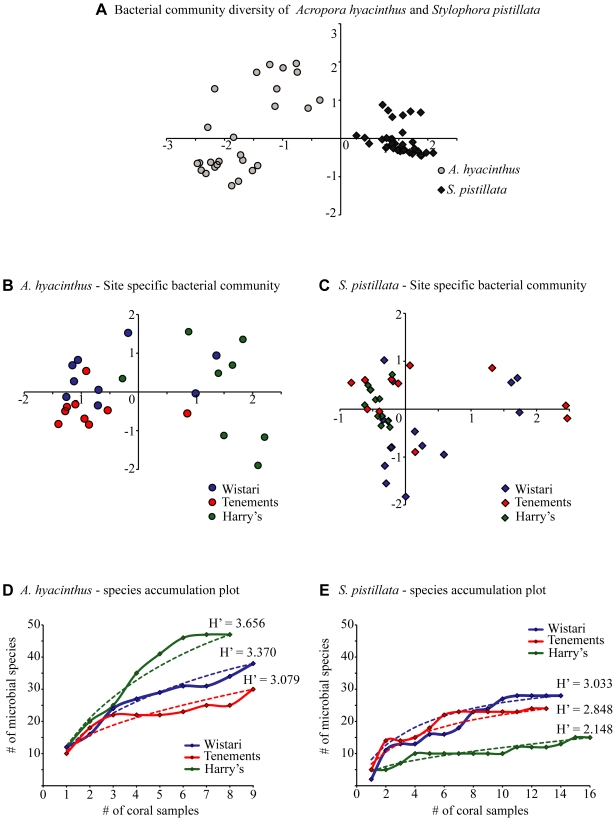
Principal components analyses (PCA) of coral bacterial communities. PCA of (A) bacterial community diversity in *Acropora hyacinthus* and *Stylophora pistillata*, (B) *A. hyacinthus* bacterial community diversity by site, (C) *S. pistillata* bacterial community diversity by site. Variation in the community structure explained by the first two PCA axes for A, B and C are 36.4%, 30.3% and 33.9%, respectively. (D, E) Species accumulation curves of *A. hyacinthus* (D) and *S. pistillata* (E) plotted for all three sites. Dotted lines represent the estimated curve based on an infinite number of samples (UGE) and the solid lines represent species accumulation based on true values (SobS). Species diversity indices (Shannon-Weaver H′) are further shown for each site. Sites: Wistari = Wistari Reef, Tenements = Tenements, Harry's = Harry's Bommie.

A total of 67 different bands were observed on DGGE profiles amongst the apparently healthy *A. hyacinthus* replicates and 38 bands were found in the apparently healthy *S. pistillata* samples. Of these 42% and 51% of the bands were unique to each species respectively. The bacterial communities of *A. hyacinthus* and *S. pistillata* were not only significantly different from each other (Figures 2, 3A; R = 0.975, p = 0.001) but also differed between sites (Figure 2; *A. hyacinthus*, R = 0.418, p = 0.01; *S. pistillata*, R = 0.212, p = 0.01). Bacterial communities associated with both coral species from Harry's Bommie, Tenements, and Wistari Reef were all significantly different from each other (*A. hyacinthus* Figure 3B; all sites differed from each other with p = <0.001: *S. pistillata* Figure 3C; Wistari vs Tenements, p = 0.035; Wistari vs. Harry's, p = 0.001; Tenements vs. Harry's, p = 0.005). Bacterial species diversity estimates for *A. hyacinthus* showed that Harry's Bommie had the highest diversity followed by Wistari Reef and Tenements (Figure 3D), whereas for *S. pistillata* Wistari Reef had the highest diversity, followed by Tenements and Harry's Bommie (Figure 3E). Species accumulation curves generated from the cumulative number of ribotypes against a measure of the sampling effort indicated that asymptote was likely achieved in *S. pistillata* (Figure 3E) thus further sampling effort would be unlikely to detect any new ribotypes. This was not achieved for *A. hyacinthus* (Figure 3D), however, suggesting further sampling of this species may potentially detect additional rare ribotypes. The species accumulation curves and the observed variations in DGGE profiles between replicate samples show that, even on small spatial scales, a sample size of less than 6 individuals per site is likely to be insufficient to explore bacterial community diversity among reef corals.

**Figure 4:**
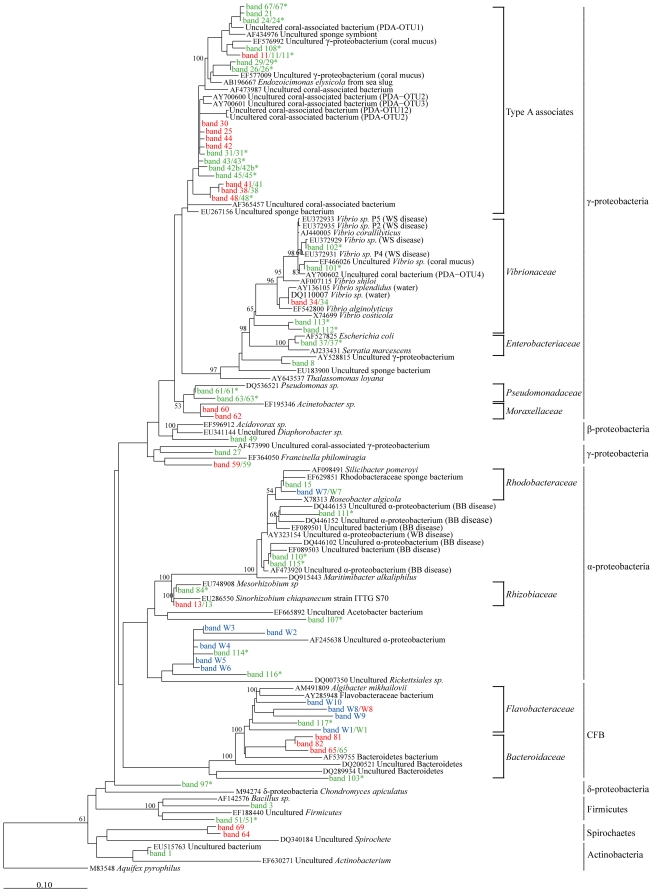
Phylogenetic analyses of obtained sequences and coral associated bacteria. Maximum likelihood consensus tree of all 16S rDNA V3 sequences obtained from denaturing gradient gel (DGGE) analyses of bacterial communities associated with *Stylophora pistillata*, *Acropora hyacinthus* and water samples. For all identified and sequenced DGGE bands, the bacterial group affiliation is shown and the taxonomic identification given to the family level where known (see Tables S1 and S2 for closest relatives). Bootstrap values were calculated based on the 1000 replicate sample sets only of the initial full length sequences used to construct the tree and only values of >50% are shown. Scale bar represents 0.1 substitutions per nucleotide position. Sequences obtained from: GenBank (black); water samples (blue); apparently healthy *S. pistillata* (red); apparently healthy *A. hyacinthus* (green), and; diseased *A. hyacinthus* (green*). GenBank accession numbers GQ924692–GQ924753 for sequences obtained in this study.

Approximately 10% of all recovered/analysed bands were abundant across samples (occurred in >50% of samples from apparently healthy colonies of one species or the other) and were coral species-specific (Figure 2A and B; Table S2A and B). Phylogenetic comparisons indicated that most of these common ribotypes were closely related and clustered as a distinct group within the gamma-proteobacteria, separate from the *Enterobacteriaceae*, *Vibrionaceae* and *Pseudomonadaceae* (Figure 4). Only one ribotype amongst this group has been named to genus and species level and represents a new genus *Endozoicimonas* (Figure 4). Moreover, previously discovered bacterial ribotypes within this distinct cluster have been found specifically associated with coral and other marine invertebrates (Figure 4). As we have insufficient information to assign the present ribotypes to a particular genus we have termed the cluster “Type A Associates” to reflect their frequent and unique association with marine invertebrates. Further ribosomal sequence and phylogenetic analyses are required before it will be possible to robustly define members of this cluster into a genus or genera.

**Figure 5:**
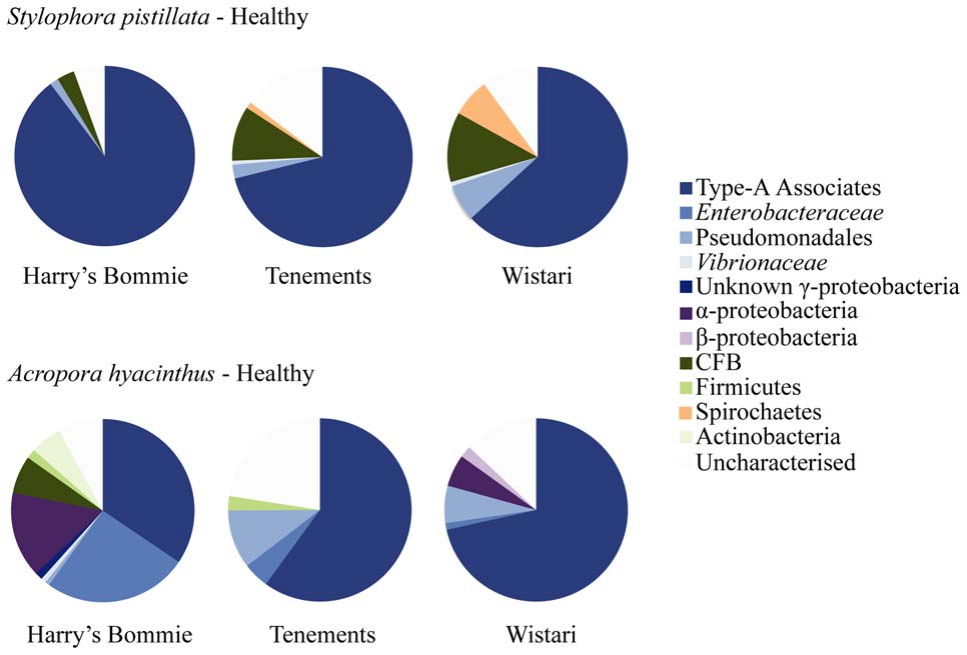
Average percent contribution of specific bacterial groups. Average percent contribution (based on SIMPER analyses results, Table S2) within *Stylophora pistillata* and *Acropora hyacinthus* at all three sites (Harry's Bommie, Tenements, Wistari Reef). γ-proteobacteria (including Type A associates, *Enterobacteraceae*, *Pseudomonadaceae*, *Vibrionaceae*) are the main constituents of the bacterial community in both species. Uncharacterised = DGGE bands from which no sequence identity was obtained were grouped; each independently contributed only a small percentage compared to the identified bands (Table S2).

In *A. hyacinthus* colonies, seven ribotypes were highly abundant (bands 31, 43, 24, 45, 42b, 26, 37; in order of average contribution). All of these belonged to the γ-proteobacteria and occurred in over 50% of colonies examined. Six of these γ-proteobacteria were identified as Type A associates and the remaining one was a member of the *Enterobacteraceae* closely related to *Escherichia coli* (Table S2C; Figure 4). Differences between the average contributions of the identified bacteria were evident by site (Table S2A, B; Figure 5), with bands 31, 42b, 43, 45 (Table S2B) occurring at higher frequency at Wistari Reef and Tenements, and the *Enterobacteraceae* ribotype (band 37) being more common at Harry's Bommie (Figure 5; Table S2B).

In *S. pistillata* four ribotypes (bands 30, 44, 81, 42; in order of average contribution) occurred in >50% of all examined colonies, three of which belonged to the γ-proteobacterial cluster termed Type A associates, and the fourth a member of the CFB group (Table S2B; Figure 4). Although bacteria from within the Type A associates cluster were found across sites, differences between the average contributions of the ribotypes were evident by site (Figure 5). For example, γ-proteobacteria Type A associate “band 42” was more abundant at Harry's Bommie than other sites, whereas a member of the CFB (band 81), and a member of the Spirochaetes (band 69) were detected more frequently in samples from Wistari Reef than other sites (Table S2A; Figure 5).

The majority of ribotypes found in both *A. hyacinthus* and *S. pistillata* occurred in <50% of all colonies examined and are therefore considered to be rare (see Table S2B, C; Figure 4 for identity). Examples in *S. pistillata* include two closely related γ-proteobacteria belonging to the family *Moraxellaceae* (band 60, 62) as well as members of the Spirochaetes group (band 64, 69) and *Flavobacteria* (band W8). Additional rare bacterial associates were also observed in *A. hyacinthus* and included members of the γ-proteobacteria, Firmicutes, β-proteobacteria, α-proteobacteria, Bacteroidetes and an Actinobacterium (band 61, 63, 3, 51, 49, 15, W7, 65, 1) (Figure 4; Table S2A, b). A member of the *Rhizobiaceae* was also prevalent in Harry's Bommie *A. hyacinthus* corals (Table S2B; Figure 2B; band 13 in samples indicated with #). Finally, a specific *Vibrio* sp. was occasionally present in apparently healthy *S. pistillata* and *A. hyacinthus* colonies (Figure 2, band 34 in samples indicated with an * in *S. pistillata* and with an ^∧^ in *A. hyacinthus*).

In samples of *A. hyacinthus* and *S. pistillata*, where the two most common closely related strains of γ-proteobacterial Type A associates (bands 31 and 30, respectively) were either missing or had very low DGGE band intensity, a dramatic shift was seen in the entire associated bacterial community (Figure 2A and B). All *S. pistillata* colonies in this category (14% of the total colonies of this species) showed a shift away from the γ-proteobacteria Type A associate (band 30) to be dominated by a *Vibrio* sp. (band 34; profiles indicated with * in Figure 2A). In these *Vibrio* sp. dominated colonies, there was also a concomitant shift towards other strain types of γ-proteobacteria than those generally associated with most healthy samples (Figure 4; sub-group containing bands 38, 41, and 48 compared to the group containing bands 30, 42, and 44). Four percent of *A. hyacinthus* colonies showed a similar shift toward the same *Vibrio* sp. as found in *S. pistillata* (band 34; profile indicated with ^∧^ in Figure 2B). The majority of *A. hyacinthus* colonies that were not dominated by the host specific strain Type A associate ribotypes (band 31) were instead dominated by either a *Rhizobiaceae* ribotype (band 13; profiles indicated with # in Figure 2B) or a mixture of three γ-proteobacterial ribotypes (bands 24, 26, 37; profiles indicated with § in Figure 2B).

### Bacterial communities of White Syndrome affected *Acropora hyacinthus*

**Figure 6:**
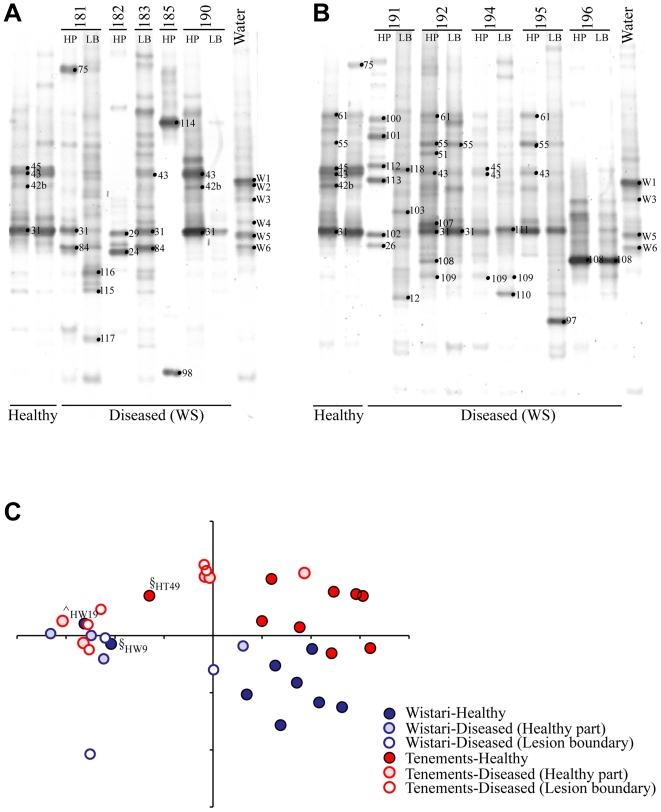
Denaturing gradient gel electrophoresis (DGGE) gels of healthy and diseased corals and water. DGGE gels of the 16S rDNA hypervariable region V3 show the bacterial communities present in White Syndrome (WS) affected (diseased) *Acropora hyacinthus* colonies from (A) Wistari Reef and (B) Tenements. The most common healthy *A. hyacinthus* and water sample DGGE profiles at each site are included as a reference. Sample numbers are shown above each lane and band numbers indicate the position of bands excised for sequence analyses. HP = healthy part of diseased colony 10 cm away from the lesion boundary; LB = tissue from the border of lesion boundary of colony (see Methods for more information). (C) Principal components analyses (PCA) of the bacterial community diversity in apparently healthy and WS diseased *A. hyacinthus*. ^∧^ ,§ identify apparently healthy samples from Figure 2, which are more similar to ‘diseased’ (WS) samples than they are to other apparently healthy samples. Variation in the community structure explained by the first two PCA axes is *27.3%*.

The bacterial communities associated with apparently healthy *A. hyacinthus* colonies differed significantly from those associated with diseased individuals (Figure 6C; R = 0.375, p = 0.001). However, no significant differences were evident between the coral tissue samples taken immediately adjacent to the disease lesion boundary and samples taken 10 cm from the lesion boundary on diseased colonies. While some bacterial ribotypes were shared between apparently healthy and diseased *A. hyacinthus* colonies (Table S2B, C; Figure 4), 24 bands were unique to ‘diseased’ colonies. No consistent pattern in presence of previously identified putative pathogens was evident amongst the bacterial communities across the diseased samples of *A. hyacinthus* colonies (i.e no one potential bacterial pathogen was consistently found in diseased samples). Rather, a range of varying colonisers from different bacterial groups not common in healthy samples were observed in the different replicate disease samples.

Members of the cluster of γ-proteobacteria termed the Type A associates group that occurred in apparently healthy colonies of *A. hyacinthus* were also identified in diseased colonies (Table S2B, C; Figure 4), except the ribotype isolated from band 108. This Type A associate was not found in apparently healthy samples and was closely related to a member of the γ-proteobacteria isolated from coral mucus (Figure 4). Similar to healthy samples, SIMPER analyses (Table S2C) indicated that the frequently occurring Type A associate γ-proteobacteria sequenced from DGGE band 31 was also present across many of the diseased samples, but in 13 out of 17 diseased samples, band 31 was either absent, present with low band intensity or shared dominance with other bacterial ribotypes (Figure 6A). Such co-occurring bacterial ribotypes included a member of the *Rhizobiaceae* (band 84) different from the one identified in apparently healthy samples (Figure 4), three α-proteobacteria (bands 110, 111 and 115) closely related to ribotypes previously isolated from corals affected with Black Band Disease in the Caribbean (Figure 4) and three α-proteobacteria (bands 114, 116, 117) closely related to bacteria isolated from the water column (Figure 4). Further ribotypes uniquely identified in diseased samples included four *Vibrio* spp. (bands 101, 102, 112 113; all of which were present in a single sample only), an unknown α-proteobacteria (band 107), a potential δ-proteobacteria (band 97), and finally a member of the CFB group (band 103). The only bands visible with high density in Figure 6A for which no sequence could be obtained were 75, 98, and 118, all appearing in single samples only.

## Discussion

This is the first study to employ such a large number of replicated samples in order to assess the bacterial communities of healthy and diseased corals, and the first culture-independent assessment of bacterial communities on Acroporid WS corals on the GBR. Despite the potential of not capturing rare or very low abundance bacterial ribotypes, the DGGE analysis used in this study indicated similar results of captured bacterial ribotypes and tentative bacterial species replacement in unhealthy corals to that of less replicated studies using other non-culture based techniques (e.g. [3], [54], [69]; Figure 4). The results from this research reinforce, with statistically relevant data, that corals harbour bacterial communities different to the water column [7], [9], [42], corroborate findings that corals associate only with certain specific bacterial groups, and that these coral-associated bacterial communities are ‘host’ species-specific [2]. The data presented here highlight a cluster of bacterial ribotypes frequently associated with corals, and the distribution of these bacterial ribotypes on healthy and diseased corals, which allows for further targeted research into a tentative link between these common coral associates and coral health. This study indicates that coral bacterial community assessments require a number of replicates per coral species and site to accurately describe the diversity present across the population and in order to draw inferences on health-related changes in the community composition. In addition, the comparisons of healthy and diseased *Acropora hyacinthus* samples showed that bacterial communities can change dramatically in diseased individuals. The DGGE profiles observed for corals displaying signs of White Syndrome comprised a range of bacterial ribotypes not generally found on healthy corals, including close relatives of bacteria previously found on Black Band Diseased corals. However the community profiles across the samples taken from diseased coral colonies were inconsistent and not indicative of a single bacterial causative agent.

### Common bacterial associates of *Acropora hyacinthus* and *Stylophora pistillata*

The coral bacterial communities identified in *A. hyacinthus* and *S. pistillata* were consistent with recent studies [2], [7], [9], [42] in that they not only differed significantly from the surrounding water column, but were also significantly different from each other. Whilst *A. hyacinthus* contained a higher diversity of bacterial associates in comparison to *S. pistillata*, members of a specific group of a cluster of γ-proteobacteria described here, dominated the bacterial communities of both coral species. Both *S. pistillata* and *A. hyacinthus* contained a closely related and coral species-specific strain of these γ-proteobacteria that was abundant across samples. These closely related strains of γ-proteobacteria were found in 86% of the *S. pistillata* colonies (band 30) and in 62% of the apparently healthy *A. hyacinthus* colonies (band 31), indicating that these bacteria were common coral-associates at the time of collection.

Using general FISH probes, γ-proteobacteria have also been found intracellularly in tissues of *Acropora* sp. and *S. pistillata* on the GBR [15] but the specific sub-group of γ-proteobacteria could not be established with the use of generalized probes. However, γ-proteobacteria closely related to the ones identified here were also found using sequence based analyses (clone libraries, tRFLP, DGGE) of *Pocillopora damicornis* and *Acropora millepora* colonies on the GBR [3], [54], *Porites astreoides*, *Diploria strigosa*, and *Montastraea annularis* colonies in the Caribbean [2], [42], and *Platygyra lamellina* colonies in the Red Sea [73] (Figure 4). It is also interesting to note that γ-proteobacteria found in over 50 samples of *Porites asteroides* (designated PA1) are closely related to the common γ-proteobacteria found in this study (Figure 4) and are speculated to constitute bacterial aggregates found within *Porites asteroides* [2], [14]. The recurrent retrieval of these closely related ribotypes, all of which fall within a particular cluster of the γ-proteobacteria, from coral specimens of different species and geographical regions suggests that this group of γ-proteobacteria occupies a specific niche possibly associated with reef coral tissues, and warrants a classification for this group. Therefore, we propose to identify this specific group as Type A coral associates until a complete taxonomic classification can be achieved.

Despite their common presence on reef corals, the function of these Type A associates is yet to be determined. However, they do cluster tightly (Figure 4) with the newly described *Endozoicomonas elysicola*, a bacterium isolated from the gastrointestinal tract of the sea slug *Elysia ornate* in Japan [74] as well as bacterial aggregates found inside the tentacular epidermis of a North Sea anemone [75]. The consistent finding of this bacterial group intracellularly in sea slugs and anemones [74], [75] and in reef building corals and sponges (Figure 4) suggests that these bacteria occur globally, associated with various benthic marine invertebrates, and warrant further investigations into their relationship with the host species.

### Rare bacterial associates of *Acropora hyacinthus* and *Stylophora pistillata*

In a limited number of *A. hyacinthus* and *S. pistillata* colonies, where the two respective dominant strains of γ-proteobacteria Type A associates were either missing or were seen as very faint bands, a dramatic shift was seen in the entire associated bacterial community. This shift occurred for most of *S. pistillata* and some of *A. hyacinthus* colonies in this category, to a community dominated by a *Vibrio* sp. (band 34) that was closely related to *Vibrio splendidus* and *Vibrio alginolyticus* (Figure 4). Other *A. hyacinthus* colonies where bands of the most commonly occurring γ-proteobacteria Type A associates were missing or faint, were instead dominated by either a *Rhizobiaceae* ribotype or showed a mixed dominance of three less common γ-proteobacteria. While this study can not conclusively rule out that PCR bias may contribute to these findings of potential species replacement, it is possible that loss of the commonly observed specific Type-A associates is linked with the appearance of other bacterial ribotypes, including members of *Vibrionaceae*. Although similar observations of putative species replacement have been reported previously in corals [54], further research into the mechanism of such a process is required before any firm conclusions can be drawn in reference to the observations in this study.

Various *Vibrio* spp. have previously been identified from corals and have been implicated as pathogens in coral diseases [40], [41]. However, *Vibrio* spp. are common marine bacteria and are also readily found on apparently healthy corals (this study; [3], [62]), which suggests a role other than a pathogen for many members of the *Vibrionaceae*. Members of both *Vibrionaceae* and *Rhizobiaceae* are involved in nitrogen fixation [76], [77] as well as the breakdown of simple amino acids. Thus, the consistent occurrence of members of these bacterial groups on apparently healthy corals may be indicative of a role in nutrient cycling in the coral holobiont.

### Site-specificity of coral-bacterial associations

The bacterial communities of both *A. hyacinthus* and *S. pistillata* colonies showed strong site specificity on small spatial scales. While site-specific bacterial differences in corals have been reported in locations affected by human induced changes in water quality [13], [53], [61], [78], studies on healthy reef systems until recently reported no spatial component to coral specific bacterial communities [2]. The study site for this research, Heron Island on the southern GBR is an offshore location with the second highest level of protection within the Great Barrier Reef Marine Park management structures and as such, fishing pressure and human influenced water quality issue are negligible and are not likely to explain the observed site-specific nature of the *A. hyacinthus* and *S. pistillata* bacterial communities. These findings are supported by observed indications of small scale spatial differences in the bacterial communities on corals in the Caribbean [68] and are in agreement with a recent study of Acroporids on the GBR that found differences in coral bacterial community structure between sites from the same reef as well as between reefs but surprisingly discovered little or no seasonal changes in these community structures [69]. Hence, small scale spatial differences in the bacterial communities on corals may be common and must be considered in sampling strategies for future studies.

The site-specific differences between coral species were most pronounced at Harry's Bommie, where colonies of *A. hyacinthus* had the most diverse bacterial communities in comparison to other sites, whereas the reverse was true for *S. pistillata* colonies (lowest diversity recorded at Harry's Bommie). Given that the same species-specific difference were not evident at the other sites it is unlikely that the single factor of differences between the coral species explains these findings. Water samples from Harry's Bommie contained *Flavobacteraceae* and *Rhodobacteraceae* ribotypes that were not present at the other sites (bands W7, W8, W9, W10). A similar trend of community shift was also observed in some healthy coral *A. hyacinthus* colonies at this site, where the host specific γ-proteobacteria Type A associate (band 31) were less dominant and instead ribotypes belonging to *Rhizobiaceae*, *Enterobacteraceae*, and *Vibrionaceae* occurred more frequently in the profiles. These three families of bacteria have been shown to occupy niches related to nutrient cycling (e.g. nitrogen-fixation, phototrophic processes, phosphate accumulation, alkane degradation; Table S2B; [76], [77], [79]). The co-incidence of site-specific differences in both water and on coral indicates that environmental conditions at Harry's Bommie may have the capacity to influence the coral associated bacterial community structure either directly or indirectly by a host response to the localised environmental conditions. Hence, local small-scale oceanographic processes may affect the bacterial communities associated with the coral colonies. These small-scale site-specific differences reiterate the necessity to analyse numerous replicates in order to establish baseline bacterial community states for healthy coral populations. Only through replicated assessment can the influence of time, space and host species be incorporated into population wide assessments of coral associated bacterial communities. Without such baseline information, our ability to explore the roles of either frequently associated bacterial ribotypes, or those associated with disease in the coral holobiont is limited.

### White Syndrome *Acropora hyacinthus*

The bacterial communities of White Syndrome (WS) affected *A. hyacinthus* colonies were significantly different from apparently healthy colonies of the same species. In most cases differences were the result of a shift in the community composition from dominance by members of the Type A associate cluster to other bacterial groups. Interestingly, Bourne et al. [54] showed that commonly occurring γ-proteobacteria (belonging to the Type A associate group) dominating healthy *Acropora millepora* colonies were replaced by *Vibrio* sp. and ribotypes related to the pathogen *Serratia marcescens* during a coral bleaching episode on the GBR. The bacterial community structure returned to the pre-bleaching Type A associate-dominated community following bleaching recovery. These observations are similar to the results observed in this study, where deviations from the most frequently observed profiles with Type A dominance are correlated with colonisation of other bacterial groups (including *Vibrio* spp.). Mucus from corals collected during bleaching contain less antimicrobial activity and putative probionts than during non-stressed times [11] and previous studies have suggested that a disruption of the bacterial community on healthy corals may provide entry niches for pathogens [17], [46], [80]. Thus, it is possible that Type A associates are correlated with coral health and further investigations into their role in the coral holobiont is highly warranted.

A recent study of WS in tabular Acroporids at Heron Island based on culture methodology identified certain *Vibrio* sp. as the causative agent of the syndrome [41]. The present study utilises, to our knowledge, the first culture independent assessment of bacterial communities on tabular Acroporid WS corals on the GBR and reveal that whilst a number of *Vibrio* ribotypes closely related to the implicated pathogen were observed in only one diseased coral sample, no previously implicated coral pathogens were found consistently across the WS affected colonies. While it cannot be ruled out that the putative pathogen may be present but undetected in most diseased samples by the method used, this seems unlikely as this method detected a range of *Vibrio* spp, including a very close relative to the previously implicated pathogen in one sample. The observed inconsistencies in colonisation of different bacterial groups in replicate diseased samples are corroborated by other studies in which the previously implicated specific bacterial pathogen for the coral disease investigated could not be detected (17, 46). Bacterial community changes resulting from the invasion of a single bacterial pathogen are expected to follow a similar succession of bacterial groups across all infected colonies, yet the results from the current study indicate a different mechanism. Using culture-independent methods, Ainsworth et al. [63] were unable to find evidence of intracellular bacterial infection as the cause of WS and showed that the symptomatic loss of coral tissue was caused by programmed cell death in the host. These data, coupled with the results from the present study, indicate that triggers of WS from non-bacterial origin should also be considered.

Finally, the bacterial communities identified in coral tissues distant from, and not incorporating the lesion area of WS-affected *A. hyacinthus* colonies were similar to communities from tissues at the lesion border, although distinct from communities found in healthy coral colonies. This finding is similar to changes observed in diseased corals in the Caribbean [17], [81] and indicative of a whole colony response whereby systemic changes originating from the coral itself are likely to underlie the cascade of changes observed in the associated bacterial community. Acroporids affected with WS around Heron Island have been shown to relocate energy away from apoptotic lesion areas [71] and, although this preserves valuable assimilates, may lead to a simultaneous disruption of the resident bacterial communities and associated loss of potential antimicrobial compounds, followed by invasion of opportunistic species, all of which are consistent with the observations on *A. hyacinthus* in this study.

## Materials and Methods

The removal of coral required for this study was approved by the Great Barrier Reef Marine Park Authority (Australian Government).

### Sample collection

Samples of apparently healthy *Acropora hyacinthus* (n = 26) and *Stylophora pistillata* (n = 43) were collected in March 2005 between 3 to 6 m of depth from each of three sites (Harry's Bommie, Tenements, Wistari Reef) around Heron Island on the southern GBR, Australia (Figure 1). Tissue samples were collected by removing fragments, 2 cm deep and 2 cm across, from the growing edge of each colony. Each colony was separated by more than 4 m to avoid sampling potential clone-mates. To prevent cross-contamination, each coral colony was sampled by snapping off a piece of coral using a new pair of disposable latex gloves and fragments placed into separate plastic bags underwater. Four replicate water samples were also collected at each site (n = 12), where 2 L of water was collected in sterile plastic zip-loc bags from approximately 10 cm above the reef structure. Samples were returned to the laboratory where the water samples were filtered immediately through a sterile 0.22 µm filter (Millipore) and preserved as for corals described below. The coral samples were first rinsed with sterilised seawater (0.22 µm filtered and autoclaved) to remove all loosely associated microbes, after which the coral tissue and associated mucus was removed by airbrushing, using sterile equipment between all samples. Both the coral and the water filter samples were suspended in 20% DMSO (final concentration) preservation buffer [82] and stored at −20°C until further processing.

During collections, White Syndrome (WS) affected colonies of *A. hyacinthus* were observed at Tenements and Wistari Reef and fragments of 10 WS affected colonies were collected at both sites in addition to the apparently healthy colonies reported above. Separate samples of the WS colonies were taken from coral tissues at the margin between tissue and bare skeleton (herein referred to as the ‘lesion boundary’), and from the part of the diseased colony approximately 10 cm from (and not incorporating) the lesion boundary (Figure 1). The respective WS samples were treated and preserved as above.

### DNA extraction, DGGE and sequencing

The DMSO-preserved samples were washed twice with DNAB buffer (0.4 M NaCl, 50 mM EDTA), and the tissue slurry homogenized with glass beads (BIO101, USA) using a MagnaLyser bead mill (Roche Diagnostics, Australia) at 3000 rpm for 90 s. DNA was extracted using a Qiagen Plant Mini Kit following the manufacturer's instructions with the inclusion of all additional steps. The universal primers 27f (5′-AGA GTT TGA TCM TGG CTC AG-3′) and 1492r (5′-TAC GGY TAC CTT GTT ACG ACT T-3′) [83] were used for an initial PCR amplification of the bacterial 16S rDNA following the standard PCR mixture recommended for Platinum *Taq* DNA polymerase (Invitrogen), with 20ng of template DNA in final reaction volumes of 50 µl, employing a ‘touchdown’ reaction protocol as follows: 94°C for 5 min (1 cycle); 94°C for 1 minute, 58-50°C annealing temperature for 1 minute, and 72°C for 2 minutes (30 cycles); 72°C for 10 min (1 cycle). The annealing temperature was decreased by 2°C every fifth cycle, from 58°C to 50°C, followed by an additional 10 cycles at 50°C.

Amplification products were purified using a QIAQuick PCR purification kit (Qiagen) and used as a template (1∶100 dilution) for a nested PCR with the internal V3 region primers 517r (5′-ATT ACC GCG GCT GCT GG-3′) and GC358f (5′-CGC CCG CCG CGC CCC GCG CCC GTC CCG CCG CCC CCG CCC CCC TAC GGG AGG CAG CAG-3′) [84]. The PCR reaction was setup following the manufacturers instructions for Ampli*Taq* Gold Polymerase (Applied Biosystems) with ∼2ng of template DNA in final reaction volumes of 100 µl, and thermocycler conditions as described above, with the exception of a change in the touchdown annealing temperature range to 63°C −55°C (with a final 10 cycles at 55°C). The 16S rDNA V3 region amplicons were screened for sequence variability using a Bio-Rad DCode denaturing gradient gel electrophoresis (DGGE) system. Between 6 to 10 µL of the PCR amplifications (∼400 ng DNA) were mixed with an equivalent volume of xylene-cyanol loading dye and loaded onto 8% acrylamide gels with an internal gradient of 25 to 60% denaturants (formamide and urea). Samples were separated by electrophoresis at 100 V for 10 h at 60°C, stained for 30 min using SYBR Green (Sigma-Aldrich, USA) and visualized using an UVIDoc digital camera gel imaging system. Control samples of known DGGE profiles were included as standards on all gels for comparative purposes and all samples were separated on replicate gels at least 3 times to ensure reproducibility and consistency of the profiles.

Due to the large number of bands identified across samples, sequencing efforts were focused on bands of specific interest (Figure 2). Such bands were those occurring across several replicate samples, bands occurring only once but with very high intensity (appearing bright in the gel images), or bands unique to diseased samples. Bands of interest were excised using sterile scalpel blades from multiple replicate (2–10) samples, which were subsequently sequenced, in order to ensure that bands of the same mobility shared the same sequence identity. DNA from the excised bands was eluted in 500 µl of dH_2_O, kept overnight at 4°C, then re-amplified using the primers 517r and either GC358f or 358f (5′-CCT ACG GGA GGC AGC AG-3′). Before sequencing, all re-amplified excised bands were re-run on DGGE to confirm that the correct band of interest was isolated. After this confirmation, PCR amplifications using the reverse (non-GC) 358f primer were purified (QIAquick PCR purification kit; Qiagen, USA) and sequenced at the Australian Genome Research Facility with the forward and reverse primers in separate reactions (BigDye Terminator V3.2 chemistry). All new sequence data was deposited in the GenBank database (http://www.ncbi.nlm.nih.gov), accession numbers GQ924692–GQ924753.

### Data and phylogenetic analysis

The DGGE banding profiles for each sample were scored with the aid of Quantity One® fragment analysis software (Bio-Rad), where a single repeatedly run sample on each gel served as a reference marker. Each band was assigned a number based on migration position in the DGGE gel and bands that were common in replicate samples (appearing at the same position in DGGE gels) were confirmed by sequence identity (from 2–10 replicate samples). All resultant band position designations were assimilated into a presence/absence matrix.

The presence/absence data of all samples were analysed using multivariate analyses (similar in approach to [3], [13], [54], [64], [65] using PRIMER-e (version 6.0; Clarke 1993). A recent comparison on multivariate analyses of presence/absence data matrices indicated that principal component analyses (PCA) provide the most robust results [85]. Thus all data assembled here were examined using PCA. To determine if coral species or sampling site had an effect on the make-up of the bacterial community, analyses of similarity (ANOSIM) were calculated based on Bray-Curtis similarity (only assigning similarity to a joined presence and not a joined absence of bands; [86]). Bacterial species diversity (Shannon-Weaver, H′) as well as species accumulation curves based on cumulative number of ribotypes collected against a measure of the sampling effort (UGE, estimated curve based on infinite sample size; and SobS, based on collected samples) were calculated based on the data for each coral species, for site and health status independently, to provide an indication whether sampling effort was sufficient to reliably represent the complete bacterial community diversity for each coral species and site. Finally, a similarity percentage (SIMPER) analyses was used to determine the relative contribution of each bacterial species to the observed similarity between the bacterial communities of each of three factors; coral species, site and health (reported percent contributions indicate the average contribution of each bacterial associate to the similarity within each grouping factor). The data presented in this study therefore does not provide quantitative analyses of the contribution of bacterial species to a single coral sample (although some suggestions are inferred from band intensity on gels) but rather represents an analysis on a population wide scale over multiple individuals.

Prior to phylogenetic analyses, forward and reverse sequences were aligned and chromatograms were analysed using Seqman software (Lasergene). All sequences were further analysed by BLAST algorithm [87] against sequences held in the GenBank database (http://www.ncbi.nlm.nih.gov/BLAST/) and the closest relatives with percent homology are used to report taxonomic affiliations to the family or group level. In addition, CHECK_CHIMERA version 2.7 (http://rdp8.cme.msu.edu/cgis/chimera.cgi) was used to check obtained sequences for chimeras produced during PCR. Phylogenetic analyses were performed with the ARB software package (http://www.arb-home.de; [88]), where initially, near full length 16S rDNA (∼1400 bp) sequences obtained from the GenBank searches were used to construct phylogenetic trees based on maximum likelihood, distance and maximum parsimony methods. *Aquifex pyrophilus* was used as an out-group. Partial sequences obtained from DGGE bands in the present study were thereafter imported to the tree whilst maintaining tree topology using the parsimony interactive tool in the ARB software. The three different algorithms produced trees with similar topology and thus only the maximum likelihood tree was used to visualise phylogenetic relationships. Bootstrap values (based on 1000 replicate data sets) were calculated for the initial tree based on near full length sequences by exporting the alignments to PAUP (version 4.02b; [89]).

## Supporting Information

Table S1 Close matches, identification, potential role (identified to closest published relatives on GenBank at the time of comparison), of the bacteria occurring in water samples collected from the three sites: Harry's Bommie, Tenements, and Wistari Reef, Great Barrier Reef.
(0.02 MB PDF)

Table S2 Close matches, identification, potential role (identified to closest published relatives on GenBank at the time of comparison), and % contribution to bacterial community structure (based on SIMPER analyses, indicating the average contribution of each bacterial ribotype to the similarity within each grouping factor) of the bacteria occurring in (A) apparently healthy *Stylophora pistillata*, (B) apparently healthy *Acropora hyacinthus*, and (C) diseased *A. hyacinthus* samples collected from the three sites: Harry's Bommie, Tenements, and Wistari Reef, Great Barrier Reef.
(0.04 MB PDF)
