# Supplementary material for: Bacterial Communities of Two Ubiquitous Great Barrier Reef Corals Reveals Both Site- and Species-Specificity of Common Bacterial Associates
Source: PLoS One. 2010 Apr 29;5(4):e10401. doi: 10.1371/journal.pone.0010401 (PMC2861602; doi:10.1371/journal.pone.0010401)
Supplement: Table S1 — Close matches, identification, potential role (identified to closest published relatives on GenBank at the time of comparison), of the bacteria occurring in water samples collected from the three sites: Harry's Bommie, Tenements, and Wistari Reef, Great Barrier Reef. (0.02 MB PDF) [file pone.0010401.s001.pdf]

Table S1. Close matches, identification, potential role (identified to closest published relatives on GenBank at the time of comparison), of the bacteria occurring in water samples collected from the three sites Harry's Bommie, Tenements, and Wistari Reef, Great Barrier Reef.

**Water associated microbes**				
**band ID**	**Group affiliation**	**Family**	**Closest relative** (% match)	**Potential role** (isolation source)
W1	CFB	*Flavobacteraceae*	EU636563 (96%)	Unknown (coral *Porites* sp., near fish farm effluent)
W2	α-proteobacteria	Undetermined	EF092739 (92%)	Unknown (marine water)
W3	α-proteobacteria	Undetermined	AM911490 (98%)	Unknown (marine water)
W4	α-proteobacteria	Undetermined	AM911490 (99%)	Unknown (marine water)
W5	α-proteobacteria	Undetermined	AM911490 (100%)	Unknown (marine water)
W6	α-proteobacteria	Undetermined	EF092542 (97%)	Unknown (marine water)
W7	α-proteobacteria	*Rhodobacteraceae*	AB262175 (99%)	Sulphur cycling (coastal bloom)
W8	CFB	*Flavobacteraceae*	AB294989 (100%)	Unknown (marine water near hot spring)
W9	CFB	*Flavobacteraceae*	EU799734 (93%)	Unknown (marine water)
W10	CFB	*Flavobacteraceae*	FJ015075 (98%)	Unknown (coral, *Pocillopora damicornis*)

CFB = *Cytophaga-Flavobacterium-Bacteroides*; band ID = DGGE band.
