# Supplementary material for: Bacterial Communities of Two Ubiquitous Great Barrier Reef Corals Reveals Both Site- and Species-Specificity of Common Bacterial Associates
Source: PLoS One. 2010 Apr 29;5(4):e10401. doi: 10.1371/journal.pone.0010401 (PMC2861602; doi:10.1371/journal.pone.0010401)
Supplement: Table S2 — Close matches, identification, potential role (identified to closest published relatives on GenBank at the time of comparison), and % contribution to bacterial community structure (based on SIMPER analyses, indicating the average contribution of each bacterial ribotype to the similarity within each grouping factor) of the bacteria occurring in (A) apparently healthy Stylophora pistillata, (B) apparently healthy Acropora hyacinthus, and (C) diseased A. hyacinthus samples collected from the three sites: Harry's Bommie, Tenements, and Wistari Reef, Great Barrier Reef. (0.04 MB PDF) [file pone.0010401.s002.pdf]

**Table S2**. **Close matches, identification, potential role (identified to closest published relatives on GenBank at the time of comparison), and % contribution to bacterial community structure (based on SIMPER analyses, indicating the average contribution of each bacterial ribotype to the similarity within each grouping factor) of the bacteria occurring in (A) apparently healthy *Stylophora pistillata,* (B) apparently healthy *Acropora hyacinthus*, and (C) diseased *A. hyacinthus* samples collected from the three sites Harry's Bommie, Tenements, and Wistari Reef, Great Barrier Reef.**

**A)** ***Stylophora pistillata***

| **band ID** | **Group affiliation** | **Family** | **Close relative** (% match) | **Potential role** (isolation source) | **% contribution** (to community similarity) | | |
|---|---|---|---|---|---|---|---|
| | | | | | **Harry's** | **Wistari** | **Tenements** |
| 11[*3] | γ-proteobacteria | Type-A Associates | EU799933 (96%) | Unknown (marine water) | - | 0.86 | - |
| 16[*1] | Unknown | - | - | - | - | 3.89 | - |
| 20 | Unknown | - | - | - | - | 0.70 | - |
| 22 | Unknown | - | - | - | - | 1.46 | - |
| 25 | γ-proteobacteria | Type-A Associates | AY700601 (98%) | Unknown (coral, *Pocillopora damicornis*) | - | 0.77 | 0.72 |
| 30 | γ-proteobacteria | Type-A Associates | AY700601 (99%) | Unknown (coral, *Pocillopora damicornis*) | 35.04 | 29.47 | 31.61 |
| 34[*1] | γ-proteobacteria | *Vibrionaceae* | DQ110007 (100%) | Potential opportunistic pathogen (marine water) | - | 0.77 | 0.72 |
| 35 | Unknown | - | - | - | 5.55 | 2.87 | 13.97 |
| 38[*1] | γ-proteobacteria | Type-A Associates | AY700600 (98%) | Unknown (coral, *Pocillopora damicornis*) | - | 2.94 | 0.72 |
| 41[*1] | γ-proteobacteria | Type-A Associates | FJ015089 (98%) | Unknown (coral, *Pocillopora damicornis*) | - | 0.25 | 0.78 |
| 42 | γ-proteobacteria | Type-A Associates | AY700601 (98%) | Unknown (coral, *Pocillopora damicornis*) | 29.20 | 8.40 | 9.67 |
| 44 | γ-proteobacteria | Type-A Associates | AY700601 (98%) | Unknown (coral, *Pocillopora damicornis*) | 25.52 | 19.68 | 26.94 |
| 48[*2] | γ-proteobacteria | Type-A Associates | AY700600 (98%) | Unknown (coral, *Pocillopora damicornis*) | - | 0.75 | 0.72 |
| 50 | Unknown | - | - | - | - | 0.25 | - |
| 60 | γ-proteobacteria | *Moraxellaceae* | EF195346 (100%) | Intestinal bacteria (moth) | 1.26 | 0.25 | 1.57 |
| 62 | γ-proteobacteria | *Moraxellaceae* | EF195346 (98%) | Intestinal bacteria (moth) | 0.28 | 6.37 | 0.90 |
| 64 | Spirocheates | Undetermined | DQ340184 (84%) | Unknown (intestinal tract mudsucker) | - | 0.72 | 1.17 |
| 69 | Spirocheates | Undetermined | DQ340184 (84%) | Unknown (intestinal tract mudsucker) | - | 6.14 | - |
| 74 | Unknown | - | - | - | - | 0.26 | 0.67 |
| 78 | Unknown | - | - | - | - | 0.25 | - |
| 80 | Unknown | - | - | - | - | 0.38 | - |
| 81 | CFB | *Bacteroidaceae* | EU636524 (89%) | Unknown (coral *Fungia* sp., near fish farm) | 3.15 | 12.54 | 4.96 |
| 82 | CFB | *Bacteroidaceae* | EU636524 (91%) | Unknown (coral *Fungia* sp., near fish farm) | - | - | 4.86 |
| W8 | CFB | *Flavobacteraceae* | AB294989 (100%) | Unknown (marine water near hot spring) | - | - | 0.20 |

| B) *Acropora hyacinthus* – Healthy samples | | | | | | | |
|---|---|---|---|---|---|---|---|
| **band ID** | **Group affiliation** | **Family** | **Close relative** (% match) | **Potential role** (isolation source) | **% contribution** (to community similarity) | | |
| | | | | | **Harry's** | **Wistari** | **Tenements** |
| 1 | Actinobacteria | Undetermined | EU515763 (100%) | Phosphate accumulation (freshwater lake) | 5.51 | - | - |
| 3 | Firmicutes | *Bacillales* | AF142576 (89%) | Potential probiotic (commercial probiotic) | 0.76 | - | - |
| 4 | Unknown | - | - | - | - | - | 0.37 |
| 8 | γ-proteobacteria | *Enterobacteraceae* | AY528815 (97%) | Alkane degradation (activated sludge) | 4.96 | 0.64 | - |
| 9*3 | Unknown | - | - | - | - | - | 1.33 |
| 10 | Unknown | - | - | - | 2.36 | - | - |
| 11*3 | γ-proteobacteria | Type-A Associates | FJ489773 (99%) | Unknown (coral, *Acropora* sp.) | 0.88 | 5.74 | - |
| 13*1 | α-proteobacteria | *Rhizobiaceae* | EU286550 (100%) | Nitrogen-fixation (Agava plant) | 15.12 | - | - |
| 14 | Unknown | - | - | - | - | - | 4.34 |
| 15*2 | α-proteobacteria | *Rhodobacteraceae* | FJ203405 (100%) | Sulphur cycling (coral, *Montastraea annularis*) | - | 5.65 | - |
| 21 | γ-proteobacteria | Type-A Associates | FJ489741 (98%) | Unknown (coral, *Acropora sp*.) | 4.87 | - | - |
| 23 | Unknown | - | - | - | - | 0.52 | - |
| 24*2 | γ-proteobacteria | Type-A Associates | FJ489741 (99%) | Unknown (coral, *Acropora sp*.) | 8.14 | 8.68 | 6.29 |
| 26*2 | γ-proteobacteria | Type-A Associates | FJ489775 (99%) | Unknown (coral, *Acropora sp*.) | 12.54 | 8.85 | 2.39 |
| 27 | γ-proteobacteria | - | EU537177 (98%) | Unknown (human skin) | 1.35 | - | - |
| 29*2 | γ-proteobacteria | Type-A Associates | FJ489775 (98%) | Unknown (coral, *Acropora sp*.) | 0.00 | 0.74 | - |
| 31*2 | γ-proteobacteria | Type-A Associates | FJ015090 (99%) | Unknown (coral, *Pocillopora damicornis*) | 4.87 | 12.69 | 12.78 |
| 32*1 | Unknown | - | - | - | 0.82 | - | - |
| 33 | Unknown | - | - | - | - | 0.52 | 0.41 |
| 34*1 | γ-proteobacteria | *Vibrionaceae* | DQ110007 (100%) | Potential opportunistic pathogen (marine water) | 0.92 | - | - |
| 36 | Unknown | - | - | - | - | 0.64 | - |
| 37*2 | γ-proteobacteria | *Enterobacteraceae* | DQ818938 (99%) | Gut microbiota (zebrafish intestinal tract) | 20.57 | 0.52 | 4.57 |
| 38*1 | γ-proteobacteria | Type-A Associates | AY700600 (98%) | Unknown (coral, *Pocillopora damicornis*) | 0.88 | - | - |
| 39*1 | Unknown | - | - | - | 0.85 | - | - |
| 40*1 | Unknown | - | - | - | - | 6.28 | - |
| 41*1 | γ-proteobacteria | Type-A Associates | FJ015089 (98%) | Unknown (coral, *Pocillopora damicornis*) | 0.85 | - | - |
| 42b*2 | γ-proteobacteria | Type-A Associates | FJ015090 (99%) | Unknown (coral, *Pocillopora damicornis*) | - | 9.46 | 12.78 |
| 43*2 | γ-proteobacteria | Type-A Associates | FJ015090 (98%) | Unknown (coral, *Pocillopora damicornis*) | 0.72 | 12.69 | 16.59 |
| 45*2 | γ-proteobacteria | Type-A Associates | FJ015090 (98%) | Unknown (coral, *Pocillopora damicornis*) | 0.72 | 12.66 | 9.18 |
| 49 | β-proteobacteria | *Comamonadaceae* | EU151530 (92%) | Diaphorobacter; nitrogen-cycler (waste water) | - | 2.07 | - |
| 51*2 | Firmicutes | Undetermined | EF188440 (97%) | Unknown (terrestrial cave) | 0.92 | 2.50 | - |
| 52*1 | Unknown | - | - | - | 0.88 | - | - |
| 53*2 | Unknown | - | - | - | 0.72 | 0.52 | - |
| 55*2 | Unknown | - | - | - | - | 3.32 | 6.57 |
| 56*2 | Unknown | - | - | - | - | 0.49 | 9.53 |

| | | | | | | | |
|---|---|---|---|---|---|---|---|
| 58[*1] | Unknown | - | - | - | - | 0.70 | - |
| 61[*2] | γ-proteobacteria | *Pseudomonadaceae* | EU434636 (100%) | Unknown (*Solanum* sp. roots) | - | 0.74 | 9.95 |
| 63[*2] | γ-proteobacteria | *Pseudomonadaceae* | AM886099 (98%) | Proteolytic activity (milk) | 0.74 | 5.86 | 0.41 |
| 65[*1] | CFB | *Bacteroidaceae* | EU636524 (92%) | Unknown (coral *Fungia* sp., near fish farm) | 6.70 | - | - |
| 76[*2] | Unknown | - | - | - | 2.36 | - | - |

**C) *Acropora hyacinthus* – Disease samples**

| **Species** (band ID) | **Species ID** (Group affiliation) | **Species ID** (Family) | **Close relative** (% match) | **Potential role** (isolation source) | **% contribution** (to community similarity) | | |
|---|---|---|---|---|---|---|---|
| | | | | | **Harrys** | **Wistari** | **Tenements** |
| 9[*3] | Unknown | - | - | - | N/A | 4.07 | - |
| 11[*3] | γ-proteobacteria | Type-A Associates | FJ489773 (99%) | Unknown (coral, *Acropora sp.*) | N/A | 2.87 | - |
| 24[*2] | γ-proteobacteria | Type-A Associates | FJ489741 (99%) | Unknown (coral, *Acropora sp.*) | N/A | - | 10.69 |
| 31[*2] | γ-proteobacteria | Type-A Associates | FJ015090 (99%) | Unknown (coral, *Pocillopora damicornis*) | N/A | 58.49 | 25.69 |
| 43[*2] | γ-proteobacteria | Type-A Associates | FJ015090 (98%) | Unknown (coral, *Pocillopora damicornis*) | N/A | 3.68 | 10.69 |
| 45[*2] | γ-proteobacteria | Type-A Associates | FJ015090 (98%) | Unknown (coral, *Pocillopora damicornis*) | N/A | 3.68 | - |
| 53[*2] | Unknown | - | - | - | N/A | 4.30 | - |
| 55[*2] | Unknown | - | - | - | N/A | - | 16.81 |
| 61[*2] | γ-proteobacteria | *Pseudomonadaceae* | EU434636 (100%) | Unknown (Solanum roots) | N/A | - | 10.69 |
| 84 | α-proteobacteria | *Rhizobiaceae* | EU083440 (98%) | Unknown (glacier soil) | N/A | 11.24 | - |
| 86 | Unknown | - | - | - | N/A | 4.30 | - |
| 92 | Unknown | - | - | - | N/A | 3.68 | - |
| 93 | Unknown | - | - | - | N/A | 3.68 | - |
| 94 | Unknown | - | - | - | N/A | - | 2.42 |
| 109 | Unknown | - | - | - | N/A | - | 13.88 |
| 108 | γ-proteobacteria | Type-A Associates | EF576992 (96%) | Unknown (Coral mucus) | N/A | - | 4.47 |
| 106 | Unknown | - | - | - | N/A | - | 3.47 |
| 110 | CFB | *Bacteroidaceae* | EU636519 (100%) | Unknown (coral Fungia sp., near fish farm | N/A | - | 1.21 |

**Bands occurring only once in diseased samples but with high intensity (see Figure 6), not contributing significantly to the similarity within diseased samples (#)**

| | | | | | |
|---|---|---|---|---|---|
| 100 | Unknown | - | - | - | # |
| 101 | γ-proteobacteria | *Vibrionaceae* | EF466026 (100%) | Potential opportunistic pathogen (coral mucus) | # |
| 102 | γ-proteobacteria | *Vibrionaceae* | EU372929 (100%) | Potential opportunistic pathogen (WS affected) | # |
| 103 | CFB | *Bacteroidaceae* | DQ289934 (86%) | Unknown | # |
| 107 | α-proteobacteria | Undetermined | EU133428 (86%) | Unknown | # |
| 111 | α-proteobacteria | Undetermined | DQ446152 (94%) | Unknown (BBD affected *Siderastrea sidereal*) | # |
| 112 | γ-proteobacteria | *Vibrionaceae* | DQ978262 (98%) | *Photobacterium* (oyster digestive tract) | # |
| 113 | γ-proteobacteria | *Vibrionaceae* | AY368537 (100%) | *Photobacterium* (marine sponge) | # |
| 114 | α-proteobacteria | Undetermined | AM911486 (98%) | Unknown (seawater) | # |

| | | | | | |
|---|---|---|---|---|---|
| 115 | α-proteobacteria | Undetermined | EU636519 (98%) | Unknown (coral *Fungia* sp., near fish farm | # |
| 116 | α-proteobacteria | - | EU917612 (92%) | Unknown (biofilm) | # |
| 117 | CFB | *Flavobacteraceae* | DQ482737 (93%) | Unknown (dinoflagellate associated bacterium) | # |

The bacteria are host species specific unless otherwise indicated: *1 occurring in apparently healthy *Stylophora* and *Acropora*; *2 occurring in apparently healthy *Stylophora* and diseased *Acropora*, or in apparently healthy *Acropora* and diseased *Acropora*; *3 occurring in apparently healthy *Stylophora* and *Acropora*, as well as diseased *Acropora*. # = bands in diseased *Acropora* samples that were only present in a single sample. CFB = *Cytophaga-Flavobacterium-Bacteroides*; band ID = DGGE band. Sequenced bands 59, 60, 62, 67 and 97 were very rare and are not included in the table as they did not contribute to the SIMPER analyses. Their phylogenetic affiliations are instead shown in Figure 4.
